# Effectiveness of dentist’s intervention in smoking cessation: A review

**DOI:** 10.4317/jced.52693

**Published:** 2016-02-01

**Authors:** Carlos Omaña-Cepeda, Enric Jané-Salas, Alberto Estrugo-Devesa, Eduardo Chimenos-Küstner, José López-López

**Affiliations:** 1 DDS. School of Dentistry. University of Los Andes, Mérida, Venezuela. Student of Master Degree in Dentistry in Oncologics and Immunocompromised Patients, School of Dentistry, Department of Odontostomatology, University of Barcelona. L’Hospitalet de Llobregat, Barcelona, Spain; 2 MD, DDS, PHD. Department of Odontostomatology. School of Dentistry. University of Barcelona. Oral Health and Masticatory System Group (Bellvitge Biomedical Research Institute) IDIBELL, University of Barcelona, L’Hospitalet de Llobregat, Barcelona 08907, Spain

## Abstract

**Introduction:**

Smoking is one of the main public health problems in developed countries. Despite extensive evidence on the effects of smoking on both oral and general health, the rate of smoking cessation is not promising.

**Material and Methods:**

To review the evidence on knowledge and programs for smoking cessation developed by dentists, a literature review was carried out on programs for smoking cessation from the dentist’s perspective, as well as a review of behavioral guidelines that have been recently proposed for these interventions. We used the keywords “Tobacco”, “Smoking Prevention”, “Public Health” AND “Dentistry”, to identify controlled studies, systematic reviews and meta-analyses published between 1999 and 2014, in Google Scholar, SCOPUS and PubMed.

**Results:**

Out of 177 studies found, 35 were considered, and these were divided into 2 groups of 20 and 15 articles respectively, according to type of study and inclusion criteria.

**Conclusions:**

There is considerable scientific evidence describing the programs for smoking cessation used in dentistry, which support their effectiveness. Overall, these are brief behavioral interventions complemented by pharmacological treatment, with the participation of the entire dental team.

** Key words:**Dentistry, nicotine, smoking cessation, tobacco.

## Introduction

The aim of this paper is to review the existing evidence on the effectiveness of programs in dental practices to help people quit smoking, as well as behavioral guidelines proposed in recent years, with the aim of extracting the information needed to establish a systematic approach that can be applied in daily dental practices, which is effective in preventing patients from smoking and helping them quit.

Smoking is one of the main public health problems in developed countries. Worldwide, tobacco kills approximately 6 million people and causes more than half a trillion dollars of economic negative impact each year (1). According to the Health Report of the Spanish Ministry of Health and Social Policy, 2009 (2), smoking related deaths in Spain fell between 40% and 50% from 1991 to 2011. The figures are still very high; in 1999 there were about 55,000 deaths among the population aged 35 and over, and approximately 49,000 deaths in 2001, which means that one in six deaths (one in four men and one in 40 women) are attributable to smoking (2-4). Currently, smoking is considered a preventable risk factor that causes most deaths in the world. Although it is known that there is a high mortality associated with smoking, it is not classified as an epidemic. The “epidemiological data” has generally been underused and is generally relegated to be of limited significance, yet we believe that it can be a useful tool for planning, managing and evaluating health policies, aimed both at prevention of smoking and to encourage smokers to quit (1,5). Tobacco has been the most dangerous preventable risk factor for over three decades, and it has a major impact on mortality in Spain (5). Over 90 % of deaths due to lip, mouth, or pharynx cancer in men over 35 in Spain in 2006 could have been avoided if the patients had not smoked (6). Smoking is also a very important etiologic factor in periodontal disease, deficits in post-operative healing and recovery, and it plays an important role in the failure of dental implants (7). This is why the dentist plays a significant role in promoting healthy lifestyles by incorporating programs for patients to help them quit smoking. It has been reported that dependence on tobacco is a chronic condition that requires repeated interventions by health care providers and multiple attempts by the patients in order to stop this habit (8,9).

## Material and Methods

In order to evaluate the effectiveness of interventions on smokers on an individual level in the biomedical field and especially in dentistry, an electronic search was conducted on the Google Scholar database, SCOPUS and PubMed (Medline) using the key-words “Tobacco”, “Smoking Prevention”, “Public Health” AND “Dentistry”, to identify controlled studies, systematic reviews and meta-analyses published between 1999 and 2014. Subsequently, these articles were subjected to the following inclusion criteria: i) Studies with statistical criteria which included samples that produced significant results; ii) Studies whose objectives were to encourage giving up smoking; iii) Studies including patients, students, support staff, public and private health services; iv) Studies on designs or comparisons of protocols that dealt with smoking in relation to dentistry; and v) articles that address and/or review behavioral therapy, nicotine replacement therapy and pharmacological studies.

## Results

**Figure 1:**
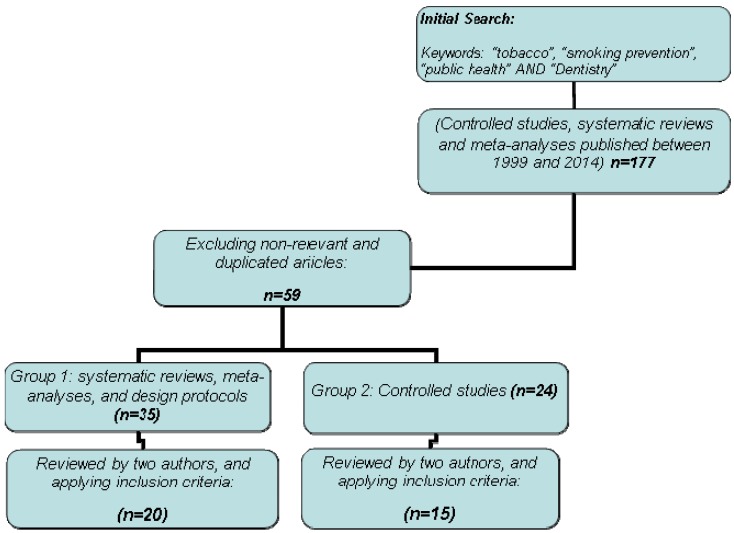
Search strategies.

Conducting a search using the described criteria, 177 articles were found (n=177), from which the most relevant articles related to our objective were chosen. This excluded duplicate publications or any articles that were not of direct interest. The inclusion of the search criteria in the abstracts and/or keywords was considered, in addition to the parameters mentioned above (n= 59). These 59 results obtained were further identified and separated into two groups: group one, with systematic reviews, meta-analyses, and design protocols (n=35), and group two, composed of controlled studies (n=24). Two different authors reviewed each group. After reviewing and applying the described criteria, 35 articles were finally included: 20 in group one and 15 in group two. (Fig. 1)

Based on the literature reviewed, the strategies most commonly used to quit smoking can be summarized as follows: *(i) behavioral therapy, (ii) nicotine replacement therapy (NRT) and (iii) pharmacological management of addiction* (10).

(i) In relation to behavioral therapy, it is suggested that dental professionals should limit themselves to brief interventions and counseling sessions with patients. Heavy smokers, particularly those with serious emotional and social problems, will require intensive behavioral intervention and should be referred to psychologists, psychiatrists, family doctors or specialists of programs to quit smoking (11). In this regard, recent studies have analyzed the effectiveness of different approaches to patients depending on the degree of dependence on tobacco and the aggressiveness of these techniques, with studies such as the one carried out by Houstonn *et al.* (12), which shows that an approach via email for smoking patients, including a variety of preventive content and oral health promotion as well as guidelines on preventing oral cancer, achieved encouraging results in the U.S. Similarly, the National Oral Health Promotion Clearing House in Australia (13) sent messages highlighting the link between smoking and various diseases, recommending giving up smoking, and encouraging patients to visit their dentist to receive further advice. Nohlert *et al.* (14) compared high-intensity interventions with low-intensity interventions in a group of patients in Sweden, regardless of their degree of addiction, in combination with other techniques. They obtained significant results for high-intensity interventions, with the low-intensity interventions varying according to behavioral support. They believed their model could be effective in helping smokers quit.

In this regard, it is worth mentioning the use of alternative therapies for behavior conditioning against the use of tobacco. It has been reported that the use of therapies such as acupuncture (15), does not show specific success rates when compared with the use of conventional techniques. Hypnosis, on the other hand, reports higher levels of success when compared to the Nicotine Replacement Therapy (16). However, larger, well-controlled, and randomized studies to assert its effectiveness are still need.

(ii) On the other hand, the objective of Nicotine Replacement Therapy (NRT) is to substitute the nicotine in tobacco, reducing the withdrawal symptoms associated with quitting smoking. NRT approximately doubled the percentage of patients who managed to quit smoking, regardless of the additional support offered (17). This is done by prescription, and the patient using different chewing gums, patches, nasal sprays, inhalers and nicotine lozenges that are available in the market. The same review concluded that all commercially available forms of NRT are effective, as they increase quitting rates by approximately 1.5 to 2 times. This effectiveness was independent of the intensity of additional support offered to the smoker. The provision of more intense levels of additional support is considered beneficial, but not essential to the success of NRT. Similarly, recent studies such as the one by Pay & Passad (10) reported that NRT is highly effective and can be combined with placebo elements. In this way, it becomes a non-invasive and non-pharmacological therapy, with excellent results when combined with counseling or mild behavioral therapy.

(iii) As for the pharmacological management of tobacco addiction, since few smokers used to succeed in giving up smoking with long-term success, a large number of pharmacological agents have been developed that have proven to double the rate of quitting, by managing the addiction as such, and treating the anxiety associated with quitting and its consequences. Most studies on the subject agree that these therapies are always more effective in conjunction with behavioral therapy (18). The most frequently used drugs to manage tobacco addiction are:

a) Bupropion, an antidepressant widely used for years for its demonstrated efficacy as a first-line drug for this purpose (18). However, current reviews suggest that use of this drug by itself (without additional therapy such as NRT) is unlikely to achieve significant clinical results (19).

b) Nortriptyline, a tricyclic antidepressant with some beneficial effects, but some side effects as well, which has comparable effects to those of Bupropion, and similar results as NRT (20).

c) Clonidine, an anti-hypertensive treatment that is widely used, and has been studied and reported to be effective in anti-smoking therapy. However, it is less frequently suggested because of its side effects, especially those related to sedation and dry mouth (21).

d) Rimonabant is a drug used to inhibit anxiety and appetite, and it is used in adjunctive treatment for obesity. Some studies show that it has beneficial effects in anxiety management of smokers in the process of quitting (18). Concerns regarding depression and suicidal ideation among rimonabant users prevented approval of rimonabant in the US and led to its suspension and eventual withdrawal in Europe (22).

e) Varenicline is an anxiolytic drug that has shown to have a significant impact in reducing smoking, with a very high cost-benefit ratio (23).

f) Finally, Cystisine is a tobacco agonist medication. It is derived from a plant extract used for decades for this purpose, and it is used to produce Varenicline. Currently, the drug has not been licensed for use due to the lack of studies on its treatment safety, but according to a meta-analysis, results are comparable to treatment with other licensed drugs, such as Varenicline, and might even be cheaper (24).

## Discussion

There is extensive and consistent evidence showing the harmful effects of smoking on oral health. This includes an association between smoking, pre-cancerous conditions and oral cancer, increased severity of periodontal disease in smokers, the negative influence of smoking on the success of certain dental treatments such as periodontal therapies, surgical treatments and placement of dental implants (7-9,25,26). Even in recent years, it has been shown that smoking might be a risk factor in certain diseases and may increase the risk of illnesses related to endodontics, which means that there is an increasing number of articles that cite the link between smoking and periapical periodontitis (in periodontal, endodontic or appearing asymptomatic teeth) (27-29).

It should not be ignored that smoking is, by far, the biggest risk factor for oral cancer. There is a strong dose-response relationship between smoking tobacco and development of pre-cancerous conditions and oral cancer (30-32). Cigarette smokers are 5 to 20 times more likely to develop oro-pharyngeal cancer than non-smokers, being the second cancer attributed to tobacco after lung cancer (31,32). Several different authors confirm that quitting smoking helps reduce the risk of oral cancer by 50% in five years. It should be noted that reducing cancer risk factors is the most effective tool in decreasing morbidity and mortality from this disease (18).

The role of dental professionals in preventing smoking and encouraging patients to quit had not been taken into account until the last 15-20 years. Several countries have developed ways to incorporate interventions against smoking in routine dental check-ups. Multiple studies, including those cited by Vendrell *et al.* (8) in 2010, indicate that training professionals is essential to implement guidelines that help patients quit smoking, since it ensures high levels of intervention and effectiveness. These studies also show that despite efforts made in recent years to involve dental staff in these tasks, few professionals include it as part of normal check-ups. Some studies highlight two key factors that limit these interventions, the lack of training for dental staff and the cost in terms of time, which leads to financial impact. That is why education for oral health professionals on this issue, with a focus on preventing oral diseases, should be considered critical to ensure the implementation and success of programs to quit smoking.

Dentists should be informed about techniques to quit smoking. We should highlight the research carried out in the United States, which trains dentists on how to implement protocols, such as the “5 As” ( Table 1). There is a direct relationship between the time spent on implementation of protocols and how well patients adhere to them (8). The same author says that if dentists could provide routine help encouraging their patients to quit smoking, even with modest success rates, the impact on public health would be enormous.

**Table 1:**
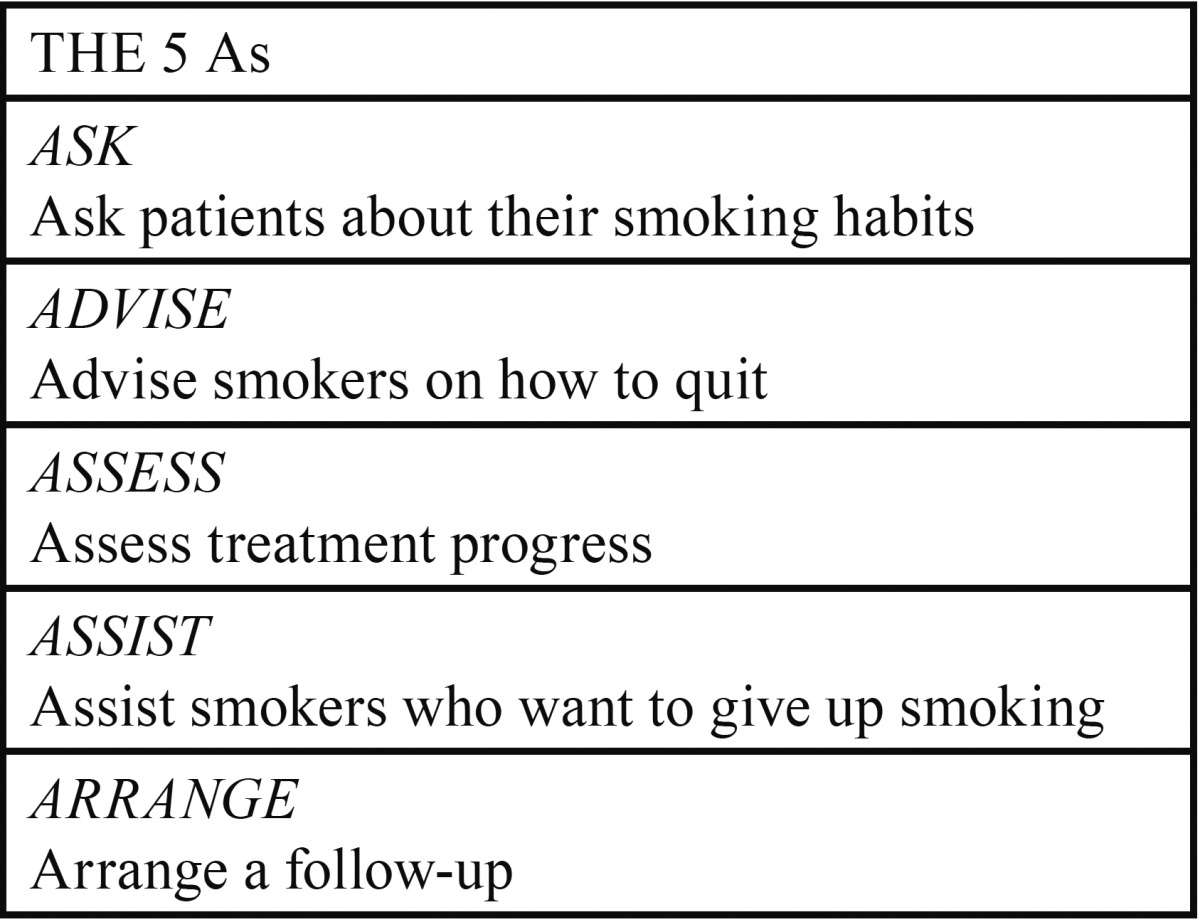
The 5 As. Taken from Vendrell *et al.* (8).

In 2010, Roseel *et al.* (34) related the reasons for dental consultation and factors that might encourage a patient to consider quitting, with no correlation shown in general terms between the reason for the check-up and any intention to quit smoking. However, clinical findings such as discoloration of teeth might change the patient’s perception on the effect smoking has on oral health, besides periodontal problems. This suggests that such issues, when present in patients who smoke, can be used as a motivational tool to quit smoking. Similarly, the perception of smoking and its effects on the oral cavity are clearly affected when diseases that are more serious occur, although recent studies suggest that the incidence of such diseases does not have a positive impact on tobacco use and smoking cessation (35).

There are a number of strategies to help people quit smoking, which can be implemented by either the dentist or dental assistants (33), personnel who are usually trained in the behavioral management of patients, and even on the pharmacological management of addiction. Several articles suggest that depending on the level of addiction, a different approach should be used, or a mix of strategies at the same time ( Table 2) (10). Despite the existing evidence and the suitability of dental clinics to discourage smoking and encourage patients to quit, knowledge and application of these approaches is very poor in daily dental practices (36). In 2003, Ramseier (37) proposed routine smoking history check-ups using a simple questionnaire, both for new patients on their first visit, and during subsequent check-ups. The questionnaire would be used as a starting point for subsequent actions. The methods used in these interventions would depend on the level of addiction, so it would be important for the questionnaire to determine whether patients have a mild (1-5 cigarettes per day), moderate (more than 10 cigarettes) or severe (more than 20 cigarettes a day) addiction (11).

**Table 2:**
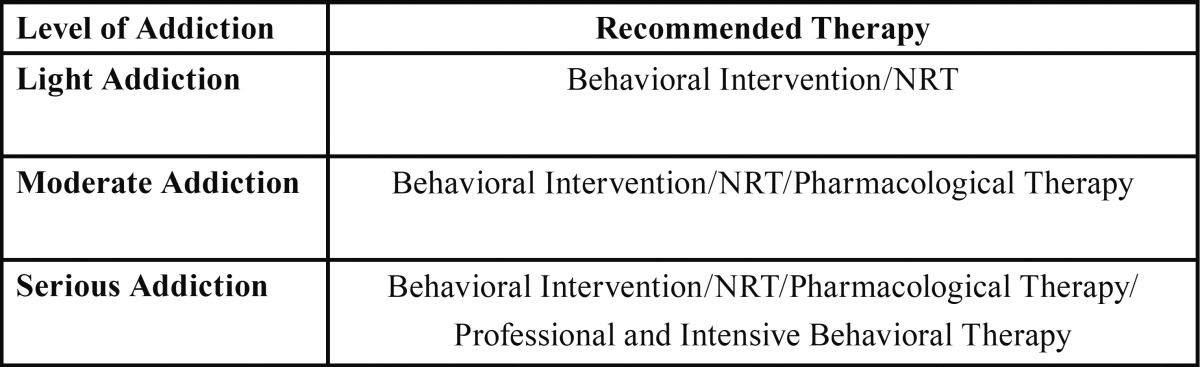
Therapy recommended quitting smoking depending on the level of addiction (10). [NRT: Nicotine Replacement Therapy].

Overall, the success rates are similar for the different types of pharmacological nicotine substitutes available. As for the decision to choose a suitable therapy, it would be based on patient-specific factors such as presence of any contraindications and side effects, as well as previous experiences with the medications, the patient’s preference and smoking history (level of addiction). The use of a combination of therapies should be reserved for cases where one approach alone was unsuccessful (18). In  Table 3, we show some of the success rates of the intervention therapies, taken from a Cochrane review (17,19).

**Table 3:**
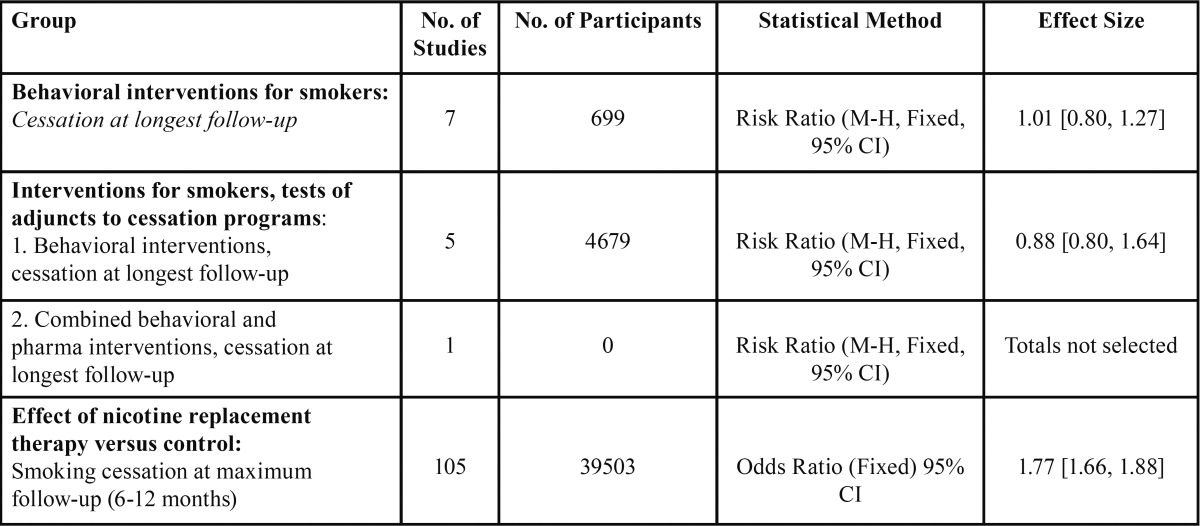
Success rates of some of the intervention therapies described. (Modified and adapted from Silagy C *et al.* (17) and Hajek P *et al.* (19), (The Cochrane Collaboration). [M-H = Mantel Haenszel statistical method] [CI = Confidence Interval].

We recommend the use of pharmacological intervention only if the patient has already failed while trying to quit smoking. It is preferable to apply the strategy of the “5 As” in combination with NRT, while pharmacological treatment should be initially avoided, depending on the level of addiction of the patient, and should always be used in conjunction with behavioral therapy, motivational counseling, and alternative therapies if patient decides so, as complementary exercise. In conclusion, dental professionals are in a unique position to identify the effects of smoking on oral health early on, and can provide tips and advice to smokers about the need to prevent or quit smoking. They should offer advice and help that is quick, simple and tailored to the patient.

Some of the studies reviewed include questionnaires, course of action, and useful advice to help develop activities for prevention, without altering the dynamics of a normal dental check-up.

In recent years the number and quality of studies on the effectiveness of these interventions in dental clinics has grown, which means it is widely accepted that the implementation of these guidelines is an effective measure with a very positive cost-benefit ratio.

The interventions on smoking habits should be considered in a comprehensive manner; personalized behavioral and alternatives therapies are necessary based on set guidelines, complemented with pharmacological therapy, if necessary.

It is important to include management strategies of tobacco cessation in the curricula of dental schools, and encourage their routine implementation.

Finally, it is important to promote studies that encourage dentists to participate in interventions to help their patients quit smoking.

## References

[1] (2014). WHO Report of the global tobacco epidemic: The MPOWER Package.

[2] (2015). Evaluación de los indicadores del estado de salud en España y su magnitud en el contexto de la Unión Europea.

[3] Saiz Martínez-Acitores I, Rubio Colavida J, Espiga López I, Alonso de la Iglesia B, Blanco Aguilar J, Cortés Mancha M (2003). National Action Plan for Prevention and Tobacco Control. Rev Esp Salud Publica.

[4] Hernández-García I, Sáenz-González M, González-Celador R (2010). Mortalidad atribuible al consumo de tabaco en España en el año 2006. An Sist Sanit Navar.

[5] Pérez-Rios M (2011). Mortalidad atribuida al consumo de tabaco: algo más que una estimación. Editorial: Med Clin (Barc).

[6] Banegas JR, Díez-Gañán L, Bañuelos-Marco B, González-Enríquez J, Villar-Álvarez F, Martín-Moreno JM (2011). La Mortalidad atribuible al consumo de tabaco en España en 2006. Med Clin (Barc).

[7] Morales-Vadillo R, Leite FP, Guevara-Canales J, Netto HD, Miranda Chaves Md, Cruz F (2013). Retrospective study of the survival and asociated risk factors of wedge shaped implants. Int J Oral Maxillofac Implants.

[8] Vendrell R, Jones D, Crews K (2010). Tobacco cessation education for dentists: an evaluation of the lecture formats. J. Cancer Educ.

[9] Myers K, Hayek P, Hinds C, Mc Robbie H (2011). Stopping Smoking Shortly Before Surgery and Postoperative Complications. A Systematic Review and Meta-analysis. Arch Intern Med.

[10] Pai A, Passad S (2012). Attempting tobacco cessation. An oral physician's perspective. Asian Pacific J Cancer Prev.

[11] Sandhu HS (2001). A practical guide to tobacco cessation in dental offices. J Can Dent Assoc.

[12] Houston TK, Coley HL, Sadasivam RS, Ray MN, Williams JH, Allison JJ (2010). DPBRN Collaborative Group. Impact of Content-Specific Email Reminders on provider Participation in an Online Intervention: A Dental PBRN Study. Stud Health Technol Inform.

[13] National Oral Health Promotion Clearing House (2011). Oral health messages for the Australian public. Findings of a natural consensus workshop. Aust Dent J.

[14] Nohlert E, Tegelberg A, Tillgren P, Johansson P, Rosenblad A, Helgason AR (2009). Comparison of a high and a low intensity smoking cessation intervention in a dentistry setting in Sweden – a randomized trial. BMC Public Health.

[15] Ming Di Y, May BH, Zhang AL, Zhou IW, Worsnop C, Xue CC (2014). A meta-analysis of ear-acupuncture, ear-acupressure and auriculotherapy for cigarette smoking cessation. Drug Alcohol Depend.

[16] Hasan FM, Zagarins SE, Pischke KM, Saiyed S, Bettencourt AM, Beal L (2014). Hypnotherapy is more effective than nicotine replacement therapy for smoking cessation: Results of a randomized controlled trial. Complement Ther Med.

[17] Silagy C, Lancaster T, Stead L, Mant D, Fowler G (2004). Nicotine replacement therapy for smoking cessation. Cochrane Database Syst Rev.

[18] Kotlyar M, Hatsukami DK (2002). Managing nicotine addiction. J Dent Educ.

[19] Hajek P, Stead LF, West R, Jarvis M, Hartmann-Boyce J, Lancaster T (2013). Relapse prevention interventions for smoking cessation. Cochrane Database Syst Rev.

[20] Hughes JR, Stead LF, Hartmann-Boyce J, Cahill K, Lancaster T (2014). Antidepresants for smoking Cessation. Cochrane Database Syst Rev.

[21] Mahvan T, Namdar R, Voorhees K, Smith PC, Ackerman W (2011). Clinical Inquiry: Which smoking cessation interventions work best?. J Fam Pract.

[22] Elrashidi MY, Ebbert JO (2014). Emerging drugs for the treatment of tobacco dependence: 2014 update. Expert Opin Emerg Drugs.

[23] Mahmoodi M, Coleman CI, Sobieraj DM (2012). Systematic review of the cost-efectiveness of varenicline vs. bupropion for smoking cessation. Int J Clin Pract.

[24] Hajek P, McRobbie H, Myers K (2013). Efficacy of cytisine in helping smokers quit: systematic review and meta-analysis. Thorax.

[25] Warnakulasuriya S (2002). Effectiveness of Tobacco Counselling in the Dental Office. J Dent Educ.

[26] Ekfeldt A, Christiansson U, Eriksson T, Lindén U, Lundqvist S, Rundcrantz T (2001). A retrospective analysis of factors associated with multiple implant failures in maxillae. Clin Oral Implants Res.

[27] López-López J, Jané-Salas E, Martín-González J, Castellanos-Cosano L, Llamas-Carreras JM, Velasco-Ortega E (2012). Tobacco smoking and radiographic periapical status: a retrospective case-control study. J Endod.

[28] López-López J, Jané-Salas E, Estrugo-Devesa A, Castellanos-Cosano L, Martín-González J, Velasco-Ortega E (2012). Frequency and distribution of root-filled teeth and apical periodontitis in an adult population of Barcelona, Spain. Int Dent J.

[29] Jiménez-Pinzón A, Segura-Egea JJ, Poyato-Ferrera M, Velasco-Ortega E, Ríos-Santos JV (2004). Prevalence of apical periodontitis and frequency of root-filled teeth in an adult Spanish population. Int Endod J.

[30] Reichart PA (2001). Identification of risk groups for oral precancer and cancer and preventive measures. Clin Oral Invest.

[31] Moreno-López LA, Esparza-Gómez GC, González-Navarro A, Cerero-Lapiedra R, González-Hernández MJ, Domínguez-Rojas V (2000). Risk of oral cancer associated with tobacco smoking, alcohol consumption and oral hygiene: a case-control study in Madrid, Spain. Oral Oncol.

[32] McCann MF, Macpherson LM, Gibson J (2000). The role of the general dental practitioner in detection and prevention of oral cancer: a review of the literature. Dent Update.

[33] Van der Waal I (2013). Are we able to reduce the mortality and morbidity of oral cancer, some considerations. Med Oral Patol Oral Cir Bucal.

[34] Rosseel JP, Hilberink SR, Jacobs JE, Maassen IM, Plasschaert AJ, Grol RP (2010). Are oral health complaints related to smoking cessation intentions?. Communit Dent Oral Epidemiol.

[35] Halawany HS, Jacob V, Abraham NB, Al-Maflehi N (2013). Oral cancer awareness and perception of tobacco use cessation counseling among dental students in four Asian countries. Asian Pacific J Cancer Prev.

[36] Amemori M, Korhonen T, Kinnunen T, Michie S, Murtomaa H (2011). Enhancing implementation of tobacco use prevention and cessation counselling guideline among dental providers: a cluster randomized controlled trial. Implement Sci.

[37] Ramseier CA (2003). Smoking prevention and cessation. Oral Health Prev Dent.

